# Tissue of origin determines cancer-associated CpG island promoter hypermethylation patterns

**DOI:** 10.1186/gb-2012-13-10-r84

**Published:** 2012-10-03

**Authors:** Duncan Sproul, Robert R Kitchen, Colm E Nestor, J Michael Dixon, Andrew H Sims, David J Harrison, Bernard H Ramsahoye, Richard R Meehan

**Affiliations:** 1 Breakthrough Breast Cancer Research Unit and Division of Pathology, University of Edinburgh, Western General Hospital, Edinburgh EH4 2XU, UK; 2 MRC Human Genetics Unit, Institute of Genetics and Molecular Medicine, University of Edinburgh, Western General Hospital, Edinburgh EH4 2XU, UK; 3 Yale University School of Medicine, Department of Molecular Biophysics & Biochemistry and Department of Psychiatry, 266 Whitney Ave, New Haven, CT 06511, USA; 4 University of St Andrews School of Medicine, Medical and Biological Sciences Building, University of St Andrews, North Haugh, St Andrews KY16 9TF, UK; 5 Centre for Molecular Medicine, Institute of Genetics and Molecular Medicine, University of Edinburgh, Western General Hospital, Edinburgh EH4 2XU, UK

## Abstract

**Background:**

Aberrant CpG island promoter DNA hypermethylation is frequently observed in cancer and is believed to contribute to tumor progression by silencing the expression of tumor suppressor genes. Previously, we observed that promoter hypermethylation in breast cancer reflects cell lineage rather than tumor progression and occurs at genes that are already repressed in a lineage-specific manner. To investigate the generality of our observation we analyzed the methylation profiles of 1,154 cancers from 7 different tissue types.

**Results:**

We find that 1,009 genes are prone to hypermethylation in these 7 types of cancer. Nearly half of these genes varied in their susceptibility to hypermethylation between different cancer types. We show that the expression status of hypermethylation prone genes in the originator tissue determines their propensity to become hypermethylated in cancer; specifically, genes that are normally repressed in a tissue are prone to hypermethylation in cancers derived from that tissue. We also show that the promoter regions of hypermethylation-prone genes are depleted of repetitive elements and that DNA sequence around the same promoters is evolutionarily conserved. We propose that these two characteristics reflect tissue-specific gene promoter architecture regulating the expression of these hypermethylation prone genes in normal tissues.

**Conclusions:**

As aberrantly hypermethylated genes are already repressed in pre-cancerous tissue, we suggest that their hypermethylation does not directly contribute to cancer development via silencing. Instead aberrant hypermethylation reflects developmental history and the perturbation of epigenetic mechanisms maintaining these repressed promoters in a hypomethylated state in normal cells.

## Background

Aberrant DNA hypermethylation of CpG island (CGI) promoters (promoter hypermethylation) occurs in many cancers. This epigenetic reprogramming is associated with the absence of transcription and can occur at a number of known tumor suppressor genes, suggesting that it contributes to tumor progression by silencing the expression of affected genes [1]. Although this model has been hugely influential, the significance of hypermethylation at CGIs in cancer has long been debated and questioned [2-4]. Also, despite intense study, the mechanisms directing promoter hypermethylation in cancer remain elusive and it is unclear whether the same mechanism operates in different cancer types. In colorectal cancer, a CGI hypermethylator phenotype (termed CIMP) has been described where hundreds of CGIs become coordinately hypermethylated during tumor progression [5,6]. Similar methylator phenotypes have been reported to occur in cancers originating from other tissues [7-9]. In these cases, it is particularly unclear whether hypermethylation is the primary event responsible for the silencing of target genes, however based on the propensity of large numbers of genes to become re-activated by exposure to DNA de-methylating drugs, it has been suggested that this might be the case [10].

Hypermethylation also plays a role in the regulation of some genes during normal development, particularly at imprinted loci and at CGI promoters on the inactive X-chromosome (Xi) in female mammalian cells [11,12]. During X-inactivation CGI hypermethylation occurs after gene silencing has already taken place [13,14] and the initial silencing event does not require DNA methyltransferases [15,16]. Absence of the maintenance methyltransferase, *Dnmt1*, in mice can lead to reactivation of the Xi later in development suggesting that in this case CGI hypermethylation acts as a stabilizing factor that maintains silencing [15]. Where the temporal dynamics of gene inactivation have been studied for autosomal genes, hypermethylation occurs subsequent to repression by other mechanisms [17].

We have recently shown that genes whose promoters are hypermethylated in breast cancer cell lines and tumors are already repressed in the putative lineage of origin and that when methylation is removed in cancer cell lines, either pharmacologically or genetically, most hypermethylated genes do not become re-activated [18]. This implies that the majority of cancer-associated CGI hypermethylation does not contribute to tumor progression under the classic model because it occurs at genes that are already switched off. Others have shown that hypermethylation of *APC *frequently occurs in gastric cancer, but at a promoter that is not utilized in normal gastric tissue [19] and that *RUNX3*, whose tumor suppressor gene status is largely based on the fact that it is frequently methylated in gastric cancer, is never expressed in the gastrointestinal epithelial cells that give rise to these tumors [20].

Here, we explore the generality of our observations in breast cancer by analyzing data derived from 1,154 tumors arising in 7 different human tissues. We show that variability in promoter CGI hypermethylation patterns between tumors is explained by variability in gene expression patterns between normal tissues and it is genes that are repressed in the pre-cancerous tissue that become preferentially hypermethylated in tumors. Our study represents the first comprehensive analysis of promoter CGI hypermethylation in different human cancers and we propose that the hypermethylation of repressed CGI promoters is a common feature of most cancers.

## Results

### Tissue of origin determines promoter hypermethylation patterns in cancers

We have previously shown that cell lineage determines promoter hypermethylation patterns in breast cancer [18]. To examine the generality of these observations in cancers arising in other tissues, we collected methylation profiling data from 1,149 tumors of 7 different cancer types: breast (Gene Expression Omnibus, [21], GEO:GSE31979), colorectal (GEO:GSE25062), prostate (GEO:GSE26126), lung (The Cancer Genome Atlas, TCGA[22]) and ovarian tumors (TCGA), along with acute-myeloid leukemias (AMLs, TCGA) and glioblastomas (TCGA) [5,8,23-25]. These datasets were all generated using Illumina Infinium HumanMethylation27 BeadChip methylation arrays, facilitating their cross comparison. We used these data to define sets of genes that were frequently aberrantly hypermethylated in each of the seven cancer types (See Additional file 1, unmethylated in the corresponding normal tissue and methylated in >20% of cancer samples, see methods for details). Our analyses were limited to genes possessing CGI promoters because the hypermethylation of non-CGI promoters is not always associated with transcriptional repression [26,27]. The number of frequently hypermethylated genes varied between cancer types with the greatest number found in colorectal and lung tumors (382 and 396 genes, respectively) and the least found in ovarian tumors (100 genes) (See Additional file 2, Figure S1A). To assess the reproducibility of these lists, we derived a second set of genes frequently aberrantly hypermethylated in breast tumors from a meta-analysis of three studies [7,18,28]. Of these 316 genes, 81.5% (256) were found in our original list, a highly significant overlap (*P *< 2 × 10^-16^, Fisher's exact test), demonstrating the reproducibility of our methodology. In total, 1,009 genes were prone to hypermethylation by this analysis in at least one type of cancer, including a number reported to be frequently hypermethylated in cancer (for example, *APC*, *DAPK1*, *ESR1*, *GSTP1*, *SFRP *genes and *HOX *genes) [29-31]. None of the 1,009 gene sets were common to all cancer types and roughly half (503 genes) were unique to a single cancer type.

**Figure 1:**
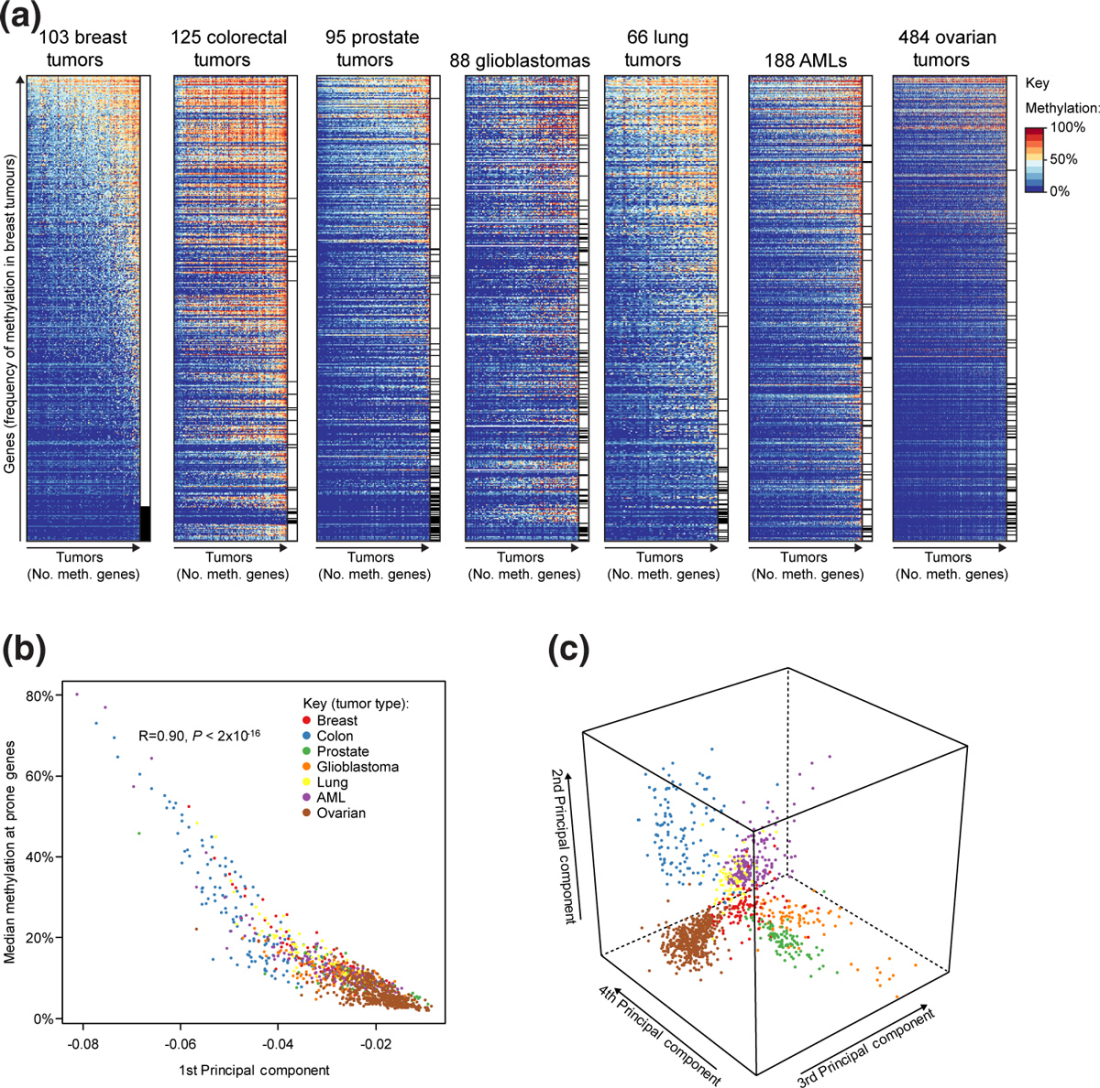
**Tissue of origin determines promoter hypermethylation patterns in cancers**. (**a**) Cancer type determines tumor methylation profiles. Shown are heatmaps of methylation levels at the 1,009 hypermethylation prone genes in 7 tumor types. Genes are ordered by their frequency of methylation in breast cancer and tumors by the number of methylated genes. The black tick marks adjacent to the heatmaps indicate genes that are never methylated in that tumor type. (**b**) Most variation between tumors corresponds to levels of methylation at hypermethylation prone genes. Shown is a scatter plot of the median methylation level at the 1,009 methylation prone genes in each of the 1,149 tumors against its value along the first principal component. Tumors are colored by type. The two values are significantly correlated (R = -0.90, *P *< 2 × 10^-16^). (**c**) Tumor type specific components exist in tumor hypermethylation patterns. Shown is a three-dimensional scatter plot of the values of each of the 1,149 tumors along the 2nd, 3rd and 4th principal components. Tumors are colored by type (as in (b)).

The overall levels of DNA methylation at these 1,009 hypermethylation-prone genes varied dramatically within cancer types but were highest in colorectal tumors and lowest in ovarian tumors (Figure 1a andAdditional file 2 Figure S1B). Examination of the methylation profiles of the 1,009 genes in the different cancer samples revealed that 220 of the genes were consistently methylated in cancers of different tissues (in at least 5% of samples for each tissue, Figure 1a). However, 446 of the genes had variable methylation profiles and were hypermethylated in some cancer types but not in others (Figure 1a, tick marks). For example, 86 of the 1,009 hypermethylation prone genes were never methylated in breast tumors but were methylated in at least one other cancer type. To systematically analyze sources of variation in the methylation profiles of the 1,149 samples, we performed principal component analysis (PCA) on the methylation data for the set of 1,009 hypermethylation prone genes [32]. The first principal component accounted for around 66% of the variance in the data and was significantly correlated with the median methylation level of the 1,009 hypermethylation prone genes (Figure 1b, R = 0.90, *P *< 2 × 10^-16^). The next three components of the data accounted for 10.4% of the variance in the data and clearly separated out the samples into the seven different tissue types (Figure 1c). These analyses indicate that a substantial number of genes are prone to hypermethylation in multiple cancer types but that the susceptibility of many other genes to hypermethylation in cancer is determined by tissue-type specific factors.

### Genes prone to hypermethylation in cancer are not constitutively expressed

**Figure 2:**
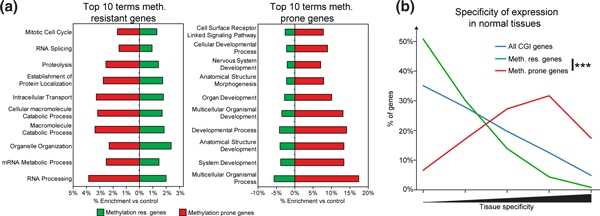
**Genes prone to hypermethylation in cancer are not constitutively expressed**. (**a**) Hypermethylation-prone and -resistant genes are associated with distinct biological processes. Shown are graphs of the percent enrichment or depletion for the 10 most enriched GO biological process in the hypermethylation resistant and prone gene sets. For each term the enrichment or depletion in both gene sets is plotted. All terms were enriched or depleted to a significant level for both gene sets (Fisher's exact tests, *P *< 0.05). (**b**) Hypermethylation prone genes are tissue-specific. Histograms show the distribution of tissue-specificity scores observed for hypermethylation prone and resistant genes. Specificity scores for prone and resistant gene sets were compared using a Wilcoxon rank sum test. (*** *P *< 0.001). GO, genome ontology.

Having defined genes that were prone to hypermethylation in cancer, we next examined which factors affected their propensity to become hypermethylated. As a control, we derived a second set of genes that were resistant to hypermethylation in cancer (those that were never methylated in any of the 1,149 cancer samples tested; 2,123 genes). The hypermethylation-prone and -resistant gene sets were associated with different Gene Ontology (GO) terms (Figure 2a). In particular, resistant genes were enriched in housekeeping terms such as 'Mitotic Cell Cycle', and 'RNA Processing and Macromolecule Catabolic Process' whereas prone genes were enriched in developmental terms such as 'System Development' and 'Organ Development'. We have previously shown that genes hypermethylated in breast cancer cell lines are expressed in a tissue-specific fashion in normal tissues and these functional terms might suggest that genes hypermethylated in diverse primary cancers also have tissue-specific expression patterns [18].

We used a method based on information theory to directly quantify the degree of tissue-specificity in a given gene's expression pattern across nine normal tissues that were profiled by high-throughput mRNA sequencing (RNA-seq, Sequence Read Archive, SRA:SRA008403) [33-35], with a higher score equating to a more tissue-specific pattern of expression. Hypermethylation prone genes were significantly more tissue-specific than hypermethylation resistant genes (Figure 2b). We observed similar results when we defined the specificity of expression from a panel of 36 tissues profiled on microarrays (See Additional file 2, Figure S2A, GEO:GSE2361) [36] or varied the thresholds used to define hypermethylation prone genes (See Additional file 2, Figure S2B). Furthermore, genes frequently hypermethylated in each of the seven different cancers were also found to have tissue-specific expression patterns (See Additional file 2, Figure S2C) as were genes found to be hypermethylated in colorectal tumors by alternative methylation profiling techniques (methyl-binding domain pull-down and sequencing, MBD-seq, or whole genome bisulfite sequencing [37-39], Additional file 2, Figure S2D, SRA:SRA029584 and [40,41]). Therefore, genes prone to hypermethylation in cancer are robustly associated with tissue-specific expression patterns in normal tissues. One possibility is that hypermethylation selectively accumulates at tissue specific genes because the disruption of many housekeeping genes might be cell-lethal. However, we found that a set of CGI promoter genes reported as recurrently mutated in breast tumors showed no preference towards either tissue specific or housekeeping expression patterns in normal tissues implying that the disruption of housekeeping genes is not necessarily lethal, at least to breast tumor cells (See Additional file 2, Figure S2E). Our analyses show that genes that are prone to hypermethylation in cancer are distinguished from those resistant to hypermethylation by their regulated expression pattern in normal tissues.

### Aberrantly hypermethylated genes have conserved promoter regions

**Figure 3:**
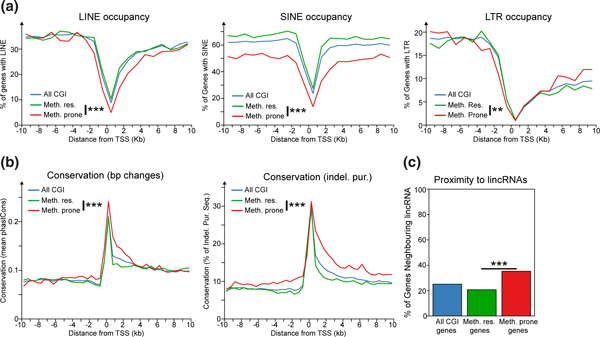
**Hypermethylated genes have conserved promoter regions**. (**a**) Hypermethylation prone promoters are depleted of repetitive elements. Shown are graphs of the frequency of LINEs, SINEs and LTRs at 1 kb intervals around hypermethylation prone and resistant TSSs. The significance of the differences in densities observed at prone and resistant genes were determined using Fisher's exact tests for the repeat counts ± 2 kb from the TSSs (*** *P *< 0.001, ** *P *< 0.01 and * *P *< 0.05). (**b**) Hypermethylation prone promoter regions are evolutionarily conserved. Shown are graphs of the level of conservation found in 500bp intervals around hypermethylation prone and resistant TSSs. Conservation was assessed through two different methods: one measuring the rate of basepair substitutions between species, 'bp Changes' [46], and the other measuring the rate of insertions and deletions between species, 'Indel. Pur.' [47]. The significance of observed differences between hypermethylation-prone and -resistant genes was assessed using a Wilcoxon rank sum test for the scores ± 2 kb from the TSSs. (**c**) Hypermethylation prone genes are found adjacent to lincRNAs. Shown is a chart of the percent of hypermethylation-prone and -resistant genes found neighboring a lincRNA [49]. The significance of differences between the gene sets was assessed using Fisher's exact tests. lincRNA, long intergenic non-coding RNAs; LTR, long terminal repeat; TSSs, transcriptional start sites.

Based on genes hypermethylated in multiple cancer cell lines, one study has suggested that the transcriptional start sites (TSSs) of genes prone to hypermethylation are depleted of repetitive elements [42]. We investigated whether this was also true of our set of hypermethylation prone genes derived from primary cancers. In our analyses, all three major classes of repetitive elements (LINEs, SINEs and long terminal repeats (LTRs)) were depleted from the TSSs of CGI promoters and to a lesser extent non-CGI promoters (See Additional file 2, Figure S3A). However, genes prone to hypermethylation in cancer had a significantly greater depletion of repetitive elements than hypermethylation resistant genes (Figure 3a). The greater depletion from the promoters of hypermethylation prone genes could be caused by an unknown activity of repetitive elements in protecting CGIs from aberrant hypermethylation as has been previously suggested [42]. However, this model is inconsistent with both the hypermethylation of repetitive elements in normal tissues and their hypomethylation in cancer [43]. Based on our observation that hypermethylation prone genes have tissue-specific expression patterns (Figure 2b), we considered an alternative scenario. The expression pattern of tissue-specific genes is often regulated by elements that lie distant to their promoter [44]. The insertion of a transposable element close to a tissue-specific gene might be detrimental to its regulation because it could directly disrupt one of these regulatory elements or interrupt their interaction with the gene promoter. The depletion of repetitive elements seen at hypermethylation prone genes could, therefore, reflect an evolutionary need to preserve the proper developmental regulation of these genes.

Many of the bioinformatic techniques used to discover functional elements in the human genome use comparisons of the genomes of multiple species to infer their presence through evolutionary conservation [45]. Therefore, a testable consequence of our hypothesis regarding the presence of regulatory elements in the vicinity of hypermethylation prone promoters is that we should detect a greater degree of evolutionary constraint or conservation around these promoters. We quantified the level of evolutionary conservation around transcription start sites using two different measures: one based on the rate of nucleotide substitutions between species [46] and the other based on the measurement of the rate of insertions and deletions between species [47]. The profiles of these scores mirrored that of repetitive elements and the greatest conservation was seen directly over the TSS (Figure 3b). Conservation was greater downstream of the TSS relative to the upstream region, probably due to the presence of exonic sequences. However, hypermethylation-prone genes had significantly higher levels of conservation as measured by both scores, at the TSS and extending into the upstream and downstream regions (Figure 3b). Similar results were observed for hypermethylation prone genes defined from either MBD-seq or whole-genome bisulfite sequencing profiling of colorectal tumors [37-39] (See Additional file 2, Figure S3B) suggesting that this property was not an artifact of data generated from Illumina Infinium arrays.

Recently, long intergenic non-coding RNAs (lincRNAs) have been proposed to play a *cis *regulatory role at some tissue specific genes [48]. Their presence is therefore a further surrogate of regulatory complexity at nearby genes, so we asked whether lincRNAs were enriched at hypermethylation prone genes. As predicted, we found that hypermethylation prone genes were significantly enriched in neighboring lincRNAs defined in a recent comprehensive analysis of human tissues when compared to hypermethylation resistant genes (Figure 3d) [49]. Thus, hypermethylation prone genes are normally expressed in a tissue-specific manner and the vicinity of their promoters is depleted of repeats and is evolutionarily conserved compared to hypermethylation resistant genes. We propose that these characteristics result from an evolutionary need to preserve regulatory elements required for the proper regulation of genes prone to hypermethylation in cancer during normal development.

### Variation in hypermethylation patterns in tumors is determined by gene expression patterns in the tissue of origin

Although repeat occupancy and conservation differ between hypermethylation-prone and -resistant genes, these factors displayed overlapping distributions for the two gene sets (See Additional file 2, Figure S3C and D). For example, some hypermethylation prone genes completely lacked SINE elements in the vicinity of their TSSs but other hypermethylation prone genes were found with more SINE elements than the average hypermethylation resistant gene (See Additional file 2, Figure S3C). Also, repeat occupancy and evolutionary conservation are invariant between different tissues and so do not explain the variable susceptibility of some genes to hypermethylation between cancers of different tissues (Figure 1a). Therefore, there must be other determinants of a gene's susceptibility to hypermethylation in a particular cancer.

**Figure 4:**
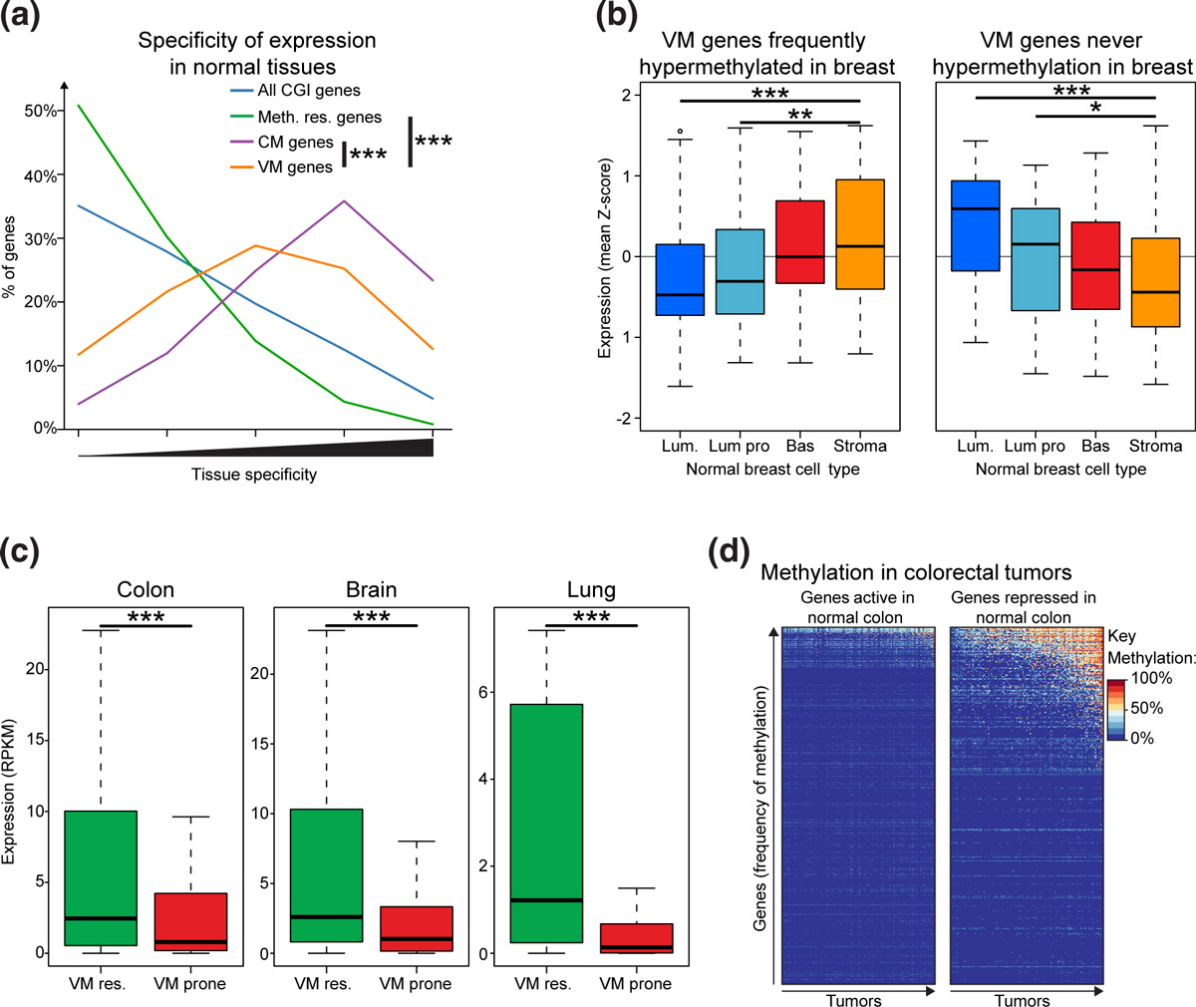
**Expression patterns in normal tissues explain differential susceptibility to hypermethylation in cancer**. (**a**) Consistently hypermethylated genes are more tissue specific than variably hypermethylated genes. Shown are histograms of tissue-specificity scores (as Figure 2b) observed at hypermethylation prone genes that were consistently or variably methylated in different tumor types. Differences between gene sets were tested using Wilcoxon rank sum tests (*** *P *< 0.001, ** *P *< 0.01 and * *P *< 0.05). (**b**) Variably hypermethylated genes with differential susceptibility in breast cancer are differentially expressed in normal breast tissue. Shown are boxplots of the relative level of expression in different cells from normal breast found at VM genes that are either frequently or never hypermethylated in breast tumors [85]. Differences between cellular fractions were tested using Wilcoxon rank sum tests. Lum = luminal epithelial cells, Lum Pro = luminal progenitor cells, Bas = basal myoepithelial cells, Stroma = breast stromal cells. (**c**) Variably hypermethylated genes that are prone to hypermethylation in tumors are repressed in the corresponding normal tissue. Shown are boxplots of the expression levels measured for VM genes with different susceptibility in individual tumor types in the corresponding normal tissues. Res = never hypermethylated in tumors, Prone = frequently hypermethylated in tumors. Differences between gene groups were tested using Wilcoxon rank sum tests. (**d**) Repressed genes are more prone to hypermethylation than active genes in colorectal cancer. Shown are heatmaps of the methylation levels of CGI promoter genes that are unmethylated in normal colon tissue and are either activated (left) or repressed (right) in normal colon as compared to normal liver. The 356 repressed genes are methylated to a significantly higher level than the 1,465 active genes (one-sided Wilcoxon rank sum test *P *= 1.6x10^-7^). CGI, CpG island; VM, variably methylated.

To uncover such determinants, we considered genes with variable methylation between tumors (VM genes, 446 hypermethylation prone genes defined as being never hypermethylated in at least one cancer type, see Additional file 3 and Figure 1a, tick marks). For comparison, we also defined a set of 220 consistently methylated (CM) genes that are methylated in all 7 cancer types (≥5% of samples of each tumor type, see Additional file 4). Both VM and CM genes were expressed in a more tissue specific fashion, depleted in repetitive elements and evolutionarily conserved compared to hypermethylation resistant genes (Figure 4a andAdditional file 2, Figure S4A and B). However, the expression of CM genes in normal tissues was significantly more tissue-specific than VM genes (Figure 4a). This suggests an inverse relationship between a gene's breadth of expression in normal tissues and the number of cancers in which it becomes hypermethylated; that is, genes that are expressed in fewer tissues become hypermethylated in more tumor types. In support of this relationship, we observed a significant correlation between a gene's specificity of expression in normal tissues and the number of tumors in which it was frequently hypermethylated (See Additional file 2, Figure S4C).

We have previously demonstrated that a gene's expression status in normal cells is linked to its susceptibility to hypermethylation in breast cancer by showing that genes repressed in a lineage-specific fashion in the normal breast are prone to hypermethylation in different subtypes of breast cancer cell lines and tumors [18]. We, therefore, examined whether gene expression patterns in normal tissues might explain the differential susceptibility to hypermethylation for VM genes in cancer. Examination of the list of VM genes along with their susceptibility suggested this might be the case. For example, *PAX6 *is prone to hypermethylation in cancer but not in glioblastomas (See Additional file 3). The gene is vital for the normal development of the brain and its expression persists into adulthood [50]. Similarly, *GFI1 *is prone to hypermethylation in cancer but not in AML and is vital for normal hematopoiesis; mice and humans lacking functional *GFI1 *are neutropenic suggesting that *GFI1 *functions in myleopoiesis and is expressed in the cells from which AMLs originate [51,52].

We tested if normal expression patterns determined hypermethylation susceptibility by considering VM genes with differential susceptibility in individual cancer types. VM genes that were frequently hypermethylated in breast tumors (67 genes) were repressed in the cells of origin of most breast tumors, luminal epithelial cells [53], as compared to normal breast stromal cells (Figure 4b, GEO:GSE16997). Conversely, VM genes that were never hypermethylated in breast tumors were active in luminal epithelial cells (Figure 4b, 86 genes). Similarly, VM genes resistant to hypermethylation in colorectal tumors, glioblastomas and lung tumors were significantly more active in the corresponding normal tissue than VM genes prone to hypermethylation in the same tumor type (Figure 4c, SRA:SRA008403), and genes that were hypermethylated in colorectal tumors, as defined by MDB-seq or whole-genome bisulfite sequencing, were also significantly less active than those that did not become hypermethylated (See Additional file 2, Figure S4D). Furthermore, expression status in normal tissues was predictive of aberrant hypermethylation in cancer as genes which were repressed in normal colon compared to normal liver were significantly more likely to be hypermethylated in colorectal tumors than genes that are active in normal colon but repressed in the normal liver (Figure 4d, one-sided Wilcoxon rank sum test *P *= 1.6 × 10^-7^, GEO:GSE13471). We have previously shown a similar preference for genes specifically repressed in luminal epithelial cells to be hypermethylated in breast tumors [18]. Together these analyses suggest that variability in promoter hypermethylation patterns between cancer types results from the variability in gene expression patterns in normal tissues, and that genes that become hypermethylated in cancer are repressed in the pre-cancerous tissue of origin.

## Discussion

**Figure 5:**
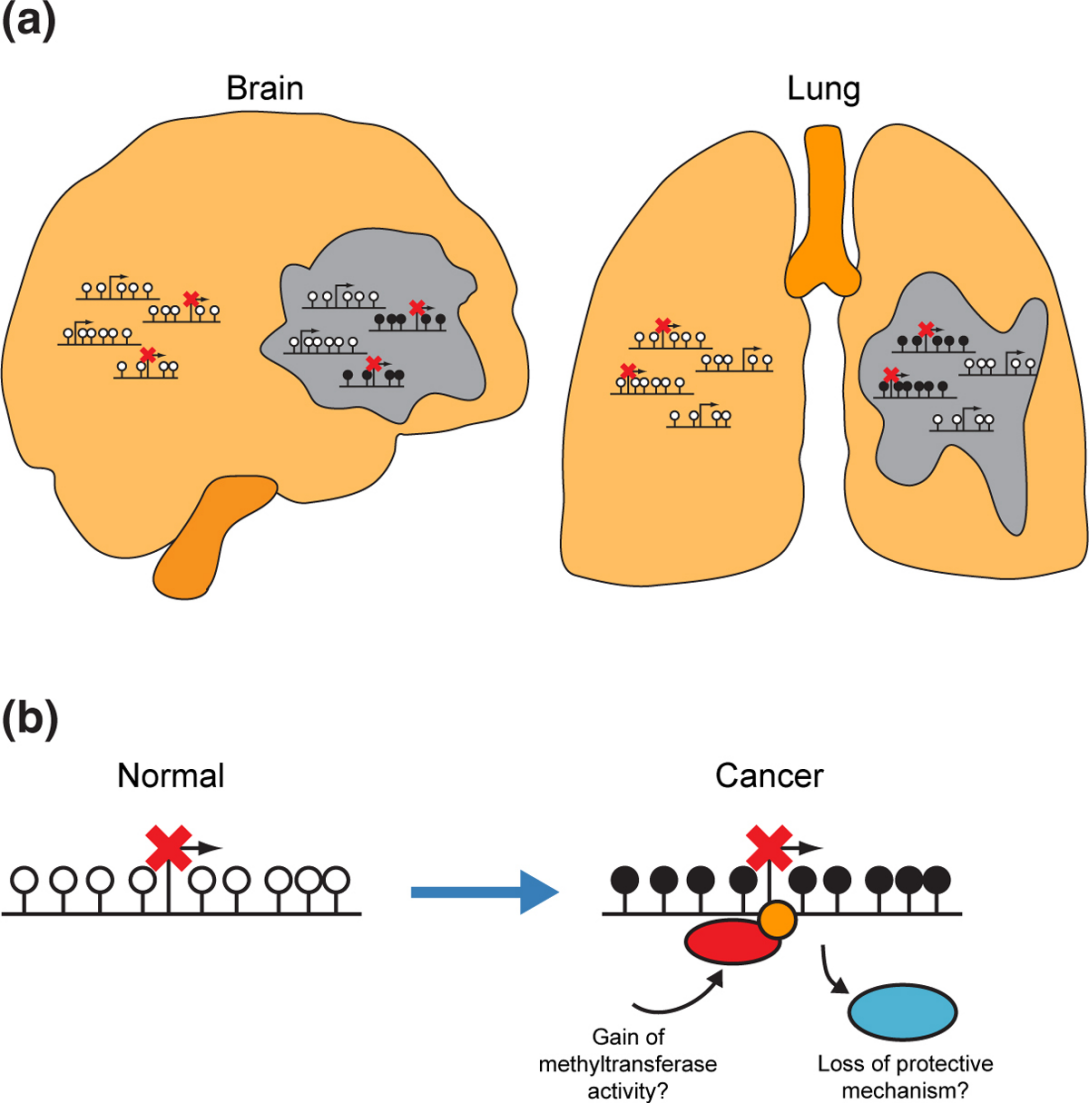
**Model: Variation in tumor hypermethylation profiles reflects gene expression in normal tissue**. (**a**) Genes repressed in a tissue-specific manner are prone to hypermethylation in tumors derived from that tissue. (**b**) Possible mechanisms that result in the hypermethylation of repressed CGI promoters in cancer. CGI promoter hypermethylation could result from either the loss of a mechanism maintaining CGIs in a hypomethylated state (for example,TET enzymes) or a gain of *de novo *methyltransferase activity at the CGI (whether targeted by transcription factors or through an increase in levels of the proteins in the cell). CGI, CpG island.

The aberrant hypermethylation of CGI promoters is of interest because it correlates with gene silencing and can occur at tumor suppressor genes [54]. Here, we present the first comprehensive analysis of CGI promoter hypermethylation in multiple cancer types and show that the genes that are hypermethylated are already repressed in the normal tissues that give rise to these tumors (Figure 5a). The potential for a gene to act as a tumor suppressor is dependent on its activity in a particular cellular context. Our study, therefore, demonstrates that the major contribution of general CGI promoter hypermethylation to cancer cannot be the silencing of tumor suppressor genes because it affects genes that are already repressed in pre-cancerous tissue.

During normal development, DNA methylation accumulates at loci that are already repressed and may facilitate stable transcriptional repression rather than directly cause silencing [55]. We have previously demonstrated that breast cancer cell lines and tumors of different lineages preferentially hypermethylate genes that are already silent in their equivalent normal cells [18]. Our current study extends this to tumors arising in different tissues and suggests that the hypermethylation of repressed genes represents a universal principle across all cancers. Other studies of individual genes also support this conclusion, for example, *RUNX3 *is frequently hypermethylated in gastric cancers but is never expressed in normal gastric epithelia [20]. Therefore, CGI promoter hypermethylation in cancer shares features with processes that occur in normal cells and does not necessarily represent a *de novo *aberrant mechanism.

Our analyses show that this model applies to the majority of hypermethylated genes found in tumors, but it has been proposed that within each tumor a few 'driver' genes are directly repressed by hypermethylation [54]. Under this scenario, the hypermethylation of repressed genes could be a 'passenger' event and is a surrogate of epigenetic dysregulation. An analogous model is proposed for genetic mutations in cancer [56,57]. Known tumor suppressor genes are hypermethylated in the tumors we analyzed but methylation of these genes generally occurs much more rarely than the hypermethylation of repressed genes, suggesting that a driver/passenger model may in fact apply (See Additional file 2, Table S1). For example, the hypermethylation of *BRCA1 *only occurs in 12% of ovarian cancers and 2% of breast cancers. We find that *APC *is hypermethylated more frequently (for example, in 33% of colorectal cancers) but it has multiple TSSs and a promoter that is repressed in normal gastric tissue has been shown to be the site of hypermethylation in gastric cancers [19]. Therefore, the significance of frequent *APC *hypermethylation depends on whether it occurs at the major promoter in these tissues. It is unclear whether the hypermethylation of these potential driver genes occurs as a by-product of the process that results in the hypermethylation of repressed genes or by an alternative mechanism (for example, the direct selection of epimutations). It is known that *MLH1 *is frequently hypermethylated in colorectal tumors that possess a CIMP phenotype [5]. However, if methylator phenotypes do generally contribute to the repression of driver genes, we would expect tumors with higher levels of promoter CGI hypermethylation to demonstrate more aggressive clinical behavior because they would be statistically more likely to have inactivated more tumor suppressor genes. Tumors with methylator phenotypes in colorectal cancer, breast cancer and glioblastoma correlate with better clinical prognosis [7,8,58].

Our results confirm a previous observation that the promoters of genes prone to aberrant hypermethylation in cancer are depleted of repetitive elements [42]. However, we suggest that this occurs due to an evolutionary need to preserve the regulation of these genes in normal development rather than as a direct protective effect of repeats as was suggested [42]. Our conclusion that hypermethylation resistant genes are primarily housekeeping genes is supported by another study that associated the presence of motifs for general transcription factors with resistance to hypermethylation in cancer [59]. The features we associate with hypermethylation prone genes overlap with those of genes regulated by Polycomb repressive complexes. For example, the prototypical gene of this class, HOX genes, are found in clusters that are devoid of repetitive elements and are regulated by lincRNAs [60,61]. Previous studies have linked promoter hypermethylation in cancer to Polycomb and the overlap between Polycomb-marked genes in embryonic stem (ES) cells and genes hypermethylated in cancer has been noted [30]. However, the profile of Polycomb marks in a single cell type is constant and does not account for the variability in hypermethylated genes between cancers of different tissues. Furthermore, although sets of hypermethylated genes are statistically enriched in these Polycomb-marked genes, only 30% to 60% of hypermethylated genes carry these marks in ES cells [5,18]. Polycomb-marked genes in ES cells carry bivalent histone modifications and are differentially activated or repressed in alternative cell lineages as differentiation proceeds [62,63]. It is therefore possible that these genes are prone to methylation because they can be repressed in a tissue-specific fashion rather than because of their association with Polycomb in ES cells.

We find that the aberrant hypermethylation of repressed genes occurs in all cancer types analyzed implying that a common mechanism might be responsible for promoter hypermethylation in all cancers. Although the exact mechanism remains unknown, our results mean that proposed mechanisms must account for the specificity of hypermethylation for repressed genes (Figure 5b). For example, if aberrant hypermethylation results from the loss of an activity protecting CGIs from hypermethylation in normal cells [3] then the specificity of hypermethylation for repressed genes implies that different factors are responsible for maintaining hypomethylation at repressed and active CGIs or that hypomethylation is maintained at active CGIs via multiple redundant mechanisms that are not all present at the CGI promoters of repressed genes. TET (ten-eleven translocation) hydroxylase enzymes may be capable of mediating this protective activity through their proposed role in DNA demethylation [64] and inhibition of their enzymatic activity in cancer correlates with the hypermethylation of CGIs [65]. Aberrant hypermethylation could also result from the recruitment of DNA methyltransferases (DNMTs) by transcription factors [66,67]. However, transcription factors also activate genes and it remains to be demonstrated how these interactions might result in the specific hypermethylation of repressed genes. Over-expression of *DNMT3B *promotes tumorigenesis in a mouse model of colorectal cancer and is associated with the hypermethylation of specific genes [68]. Higher DNMT3B levels have also been associated with the CIMP phenotype in human colorectal tumors [69,70]. One of these studies also determined the stage in tumorigenesis at which different genes became hypermethylated showing that the repressed gene *RUNX3 *was the earliest CGI promoter to show significant change [20,69], suggesting that differences in the expression of DNMTs could be linked to the hypermethylation of repressed genes.

Here we have shown that differences exist in the aberrant hypermethylation profiles of cancers arising in different tissue contexts. However, our results also make it clear that there is heterogeneity in the methylation profiles within particular types of cancer (Figure 1a). It is unclear how this heterogeneity arises but some mutations may play a direct role in its generation, for example, those that inhibit TET enzyme activity [8,65]. Colorectal cancer has previously been split into at least three groups based on methylation profiles: non-CIMP tumors, CIMP-high tumors associated with *BRAF *mutations and CIMP-low mutations associated with *KRAS *mutations [5,71]. Interestingly, a recent study suggested that CIMP-low tumors hypermethylate a subset of the genes hypermethylated in CIMP-high tumors rather than distinct sets of genes [5]. Our own results might also suggest that variation between cancers in a given tissue can manifest itself as variable levels of methylation at methylation prone genes rather than the hypermethylation of alternative gene sets (see Figure 1a). We have previously shown that differences in the hypermethylation profiles of breast cancer subtypes of putatively different cells of origin can arise because of differences in gene expression in normal cell populations [18]. Taken together, these results suggest that the hypermethylation of genes that are repressed in the normal cells of origin can account for the heterogeneity of tumor methylation profiles and variation in aberrant hypermethylation arises due to variations in the cells of origin or other factors, such as mutations, that influence the strength of the repressed gene methylator phenotype.

Recently, hydroxymethylated cytosine (hmC) has been re-discovered as a DNA modification present at significant levels in mammalian cells [72]. The Illumina arrays that were used to generate most of the datasets we have analyzed are unable to distinguish methylated cytosine (mC) from hmC [73] and the results we present may relate to hmC rather than mC marked promoters in cancer. However, we have confirmed that these results equally apply in additional datasets derived by MBD pull-down, which is specific for 5mC (See Additional file 2, Figures S2D, S3B and S4D). In addition, hmC appears to be generally depleted in cancer [74-76]. It is likely, therefore, that repressed genes are prone to hypermethylation rather than hyperhydroxymethylation.

## Conclusions

In summary, our results argue that the bulk of aberrant promoter hypermethylation in cancer occurs predominantly at genes that are repressed in pre-cancerous tissue and therefore does not directly contribute to tumor progression by silencing tumor suppressor genes. This epigenetic alteration is common to all the cancer types we have analyzed implying that a common mechanism is responsible for promoter hypermethylation at repressed genes in all cancers. Future research in this field should, therefore, focus on confirming whether aberrant hypermethylation does directly suppress rare driver genes and if the mechanism responsible for driver gene suppression is the same as that acting at repressed genes. Finally, we would suggest that researchers must exercise caution in assigning a tumor suppressor status to a gene based on its propensity to become hypermethylated in cancer.

## Materials and methods

### Statistical analyses

All statistical analyses were performed using the R statistical software (version 2.12.1) [77]. Additional packages used are mentioned under the appropriate section.

### Data sources

Gene expression and methylation data used in this study were taken from previously published studies. The sources of the data are indicated in Table 1 and the number of samples in each dataset in Table 2.

**Table 1:** Sources of methylation and expression data.

Tissue Type	Reference	Data Source	Type
Breast Tumor	[23]	GEO (GSE31979)	Methylation (Inf 27k)
Colorectal Tumor	[5]	GEO (GSE25062)	Methylation (Inf 27k)
Prostate Tumor	[24]	GEO (GSE26126)	Methylation (Inf 27k)
Glioblastoma	[8]	TCGA [22]	Methylation (Inf 27k)
Lung Tumors	[22]	TCGA [22]	Methylation (Inf 27k)
AML	[22]	TCGA [22]	Methylation (Inf 27k)
Ovarian Tumors	[25]	TCGA [22]	Methylation (Inf 27k)
Normal Tissues	[18]	GEO (GSE26990)	Methylation (Inf 27k)
Normal Tissues	[81]	GEO (GSE30090)	Methylation (Inf 27k)
Colorectal Tumors	[38]	Publication Supplementary Dataset S1 [40]	Methylation (MBD-seq)
Colorectal Tumors	[39]	SRA (SRA029584)	Methylation (MBD-seq)
Colorectal Tumor	[37]	Author's Website [41]	Methylation (WG bis-seq)
Breast Tumors	[7]	GEO (GSE26349)	Methylation (Inf 27k)
Breast Tumors	[18]	GEO (GSE26990)	Methylation (Inf 27k)
Breast Tumors	[28]	Author's Website [82]	Methylation (Inf 27k)
Normal Tissues	[36]	GEO (GSE2361)	Expression (Affy 133A)
Normal Tissues	[34]	SRA (SRA008403)	Expression (RNA-seq)
Normal Breast Tissue Cell Fractions	[85]	GEO (GSE16997)	Expression (Ill v3)
Normal Colon and Liver	[89]	GEO (GSE13471)	Expression (Affy 133plus2)

Details of data sources used in each study. The tissue type found in each dataset, the study this data were taken from, the data type and the source of the original data used are indicated. GEO and SRA accession numbers are included in the table along with links to data from other sources. Inf 27k, Illumina Infinium HumanMethylation27 BeadChip array; MBD-seq, Methyl Binding Domain pull-down followed by sequencing; WG bis-seq, whole genome bisulfite sequencing; Affy 133A, Affymetrix U133A genechip; Affy 133plus2, Affymetrix U133plus2 genechip; Ill v3,Illumina WG6 V3.0 human beadchips. Data from Noushmehr *et al*. 2011 (Glioblastoma methylation)[8] and TCGA 2011 (Ovarian tumor methylation)[25] were limited to IDs identified in these publications when downloaded from the TCGA website[22]. Methylation data from lung tumors and AML were downloaded from the TCGA website on 19 October 2011. AML, acute myeloid leukemia.

**Table 2:** Dataset sample numbers for cancer methylation data.

Tissue Type	Number of Cancer Samples	Number of Normal Samples	Technology
Fackler Breast Tumors	103	21	Infinium 27k
Fang Breast Tumors	39	2	Infinium 27k
Sproul Breast Tumors	34	2	Infinium 27k
Van der Auwera Breast Tumors	62	10	Infinium 27k
Colorectal Tumors	125	29	Infinium 27k
Illingworth Col. Tum.	5	5	MDB-seq
Xu Col. Tum.	6	3	MDB-seq
Berman Col. Tum.	1	1	WG bis-seq
Prostate Tumors	95	86	Infinium 27k
Glioblastomas	88	2	Infinium 27k
Lung Tumors	66	24	Infinium 27k
AMLs	188	8	Infinium 27k
Ovarian Tumors	484	8	Infinium 27k

Details of the number of methylation profiles analyzed for each cancer type. The cancer type, the number of cancer and normal samples analyzed and the technology used to generate the methylation profiles in each case are shown. AMLs, acute myeloid leukemias; Col. Tum, colorectal tumors; Infinium 27k, Illumina infinium Infinium HumanMethylation27 BeadChip array; MBD-seq, Methyl Binding Domain pull-down followed by sequencing; WG bis-seq, whole genome bisulfite sequencing.

### Genome annotation

In order to apply a consistent annotation to the data used in this study, all data were re-annotated to Ensembl 54 gene IDs (NCBI36). CpG probes from the Illumina Infinium arrays were mapped to the closest Ensembl gene based on TSS location using custom Perl and R scripts. CpGs that ambiguously mapped to more than one gene ID were removed from the analysis. CGI locations were taken from those biologically defined in a recent study [38]. Similarly, expression data were mapped as previously described for Illumina expression arrays [18] or using publically available re-annotations for Affymetrix expression arrays [78]. RNAseq data were mapped to Ensembl gene IDs as described below. lincRNAs were mapped to neighboring Ensembl gene IDs as described below.

### Processing of methylation data

For data originating from Infinium methylation arrays, beta values were used as a measure of the methylation level at a given CpG probe (derived from the intensity of the methylated, *I_meth_*, and unmethylated, *I_unmeth_*, allele probes: *I_meth _*/(*I_meth _*+ *I_unmeth_*) ). We have previously shown that these are a reliable estimate of the level of methylation at a locus [18]. These data were then filtered to remove unreliable values based on the detection *P*-value from the Infinium arrays (threshold 0.01). Methylation data originating from other techniques (MDB-seq or whole-genome bisulfite sequencing) were either downloaded as processed data provided by the authors [37,38] or processed from raw sequencing files [39]. We first downloaded raw sequencing data from the SRA [35]. We then aligned these reads to the genome using Bowtie (version 0.12.7) [79] and the BEDtools (version 2.12.0) *coverageBED *tool to quantify the number of reads at each CGI [80]. The read counts of CGIs were then normalized for CGI length and the total number of reads per sample to obtain a reads per kb per million mapped reads (RPKM) value for each CGI and the mean value taken from replicates of individual samples.

### Definition of hypermethylation-prone and -resistant genes

Hypermethylation-prone and -resistant genes were defined from Illumina infinium array data using beta value cutoffs (roughly equating to percent methylation divided by 100). Previously, we have shown that probes with beta values <0.3 represent unmethylated areas of the genome [18] and we therefore defined unmethylated probes on this basis. In cell lines, we have previously shown that probes with beta >0.7 represent genomic loci that are fully methylated [18]. However, in a preliminary analysis, we found that in the breast tumor samples used here, probes that had beta values >0.7 were also all methylated in normal breast tissue (data not shown). Probes that were aberrantly hypermethylated in these tumors had lower beta values because of the mix of cancerous and normal tissue in the samples analyzed. In this study, we therefore set a beta value threshold of >0.3 to define methylated probes. We only considered probes that were located within a CGI and within 200bp of a TSS that were unmethylated in all available normal samples from that tissue when defining gene sets (the 'all' genes control set for each tumor type). Frequently hypermethylated genes for each cancer were defined as genes satisfying these criteria that were methylated in at least 20% of tumor samples. Similarly, hypermethylation resistant genes satisfied these criteria but were not found to be methylated in any of the tumors. Genes present in both lists were then excluded from the analysis as being of ambiguous status to control for the presence of multiple probes at some genes.

To ensure that the method of gene selection did not bias our results, we also carried out analyses in which parameters were varied (See Additional file 2, Figure S2B and data not shown). We considered two major variations: we varied the threshold used to define aberrantly methylated genes and we varied the threshold required to call genes frequently aberrantly hypermethylated. In the first case, aberrantly hypermethylated genes were defined as those for which no probes had beta >0.3 in normal tissue and for which their mean beta value was >0.5 in at least 20% of cancers of a given type. In the second case, we varied the percent of samples required for a gene to be defined as frequently hypermethylated from 10% to 50%.

Two of the datasets used did not contain normal samples to define probes' normal tissue methylation status. In these cases, we made use of other datasets. For glioblastoma we used the fetal and adult brain samples from Sproul *et al*. [18]. For AML we used the whole blood, neutrophils, B-cells, CD4 and CD8 T-cells, natural killer cells and CD34^+ ^hematopoeitic stem cells samples from Calvanese *et al*. [81]. We then defined the 1,009 hypermethylation prone genes from those being frequently hypermethylated in at least 1 of the 7 cancers, and the 2,123 hypermethylation resistant genes as those that were never methylated in any of the tumors analyzed. Consistently and variably prone genes (CM and VM, respectively) were defined as hypermethylation prone genes that were methylated in at least 5% of tumors of each type or never methylated in at least one tumor type, respectively.

To validate the reproducibility of our method of defining hypermethylation prone genes, we compared our list of genes frequently hypermethylated in breast tumors to a second list defined by the cross-comparison of three independent studies [7,18,28]. Data from these studies were either downloaded from GEO or from the author's website (GEO: GSE26349 and GSE26990) [82]. Frequently hypermethylated genes were defined as above but only genes that were frequently hypermethylated in all three datasets were included in the analysis.

To define frequently hypermethylated and resistant genes from the Illingworth *et al*. MBD-seq data, we first generated lists of CGIs that were unmethylated in all of the normal colon samples [38]. We then defined those CGIs that had higher levels of methylation in at least two of the tumor samples when compared to their matched normal samples as frequently hypermethylated CGIs. Resistant CGIs were defined as those that did not show higher levels of methylation in any of the tumors compared to their matched normal tissues. CGIs were assigned to genes if their transcription start site was present in the CGI. Genes present in both frequent and resistant lists were also removed because their status was ambiguous.

To define genes which were hypermethylated in colorectal tumors from the Xu *et al*. MDB-seq data [39], we used one-sided Wilcoxon rank sum tests to find CGIs with significantly more reads in tumor samples than normal samples (*P *< 0.05). CGIs were assigned to genes if their TSS was present in the CGI. Using this methodology, we were unable to define a set of hypermethylation resistant genes.

We defined genes prone to and resistant to hypermethylation from the Berman *et al*. whole- genome bisulfite sequencing data [37] as genes with CGI TSSs which were located in regions defined as methylation-prone or -resistant in that study. These regions were downloaded from the author's website. Genes which were defined as both methylation-prone and -resistant were excluded as being of ambiguous status.

### Processing of expression data

To process RNA-seq data, raw sequence data for nine human tissues [34] were downloaded from GEO and converted to FASTQ format using the SRA Toolkit (version 2.1.7). Several technical replicates were available for each tissue. However, we randomly chose a single replicate in each case for simplicity and because different tissues had different numbers of replicates in this dataset. We examined each sample for per-base and per-read quality and over-represented kmers using the FastQC software (version 0.9.4) [83]. Reads were mapped simultaneously to the human genome (NCBI version 36/hg18) and a library containing the sequences of all possible exon splice junctions (Ensembl 54 exons) created using RSEQtools [84]. Reads were mapped using Bowtie (version 0.12.7) [79] allowing for a maximum of two mismatched bases and reporting the single best alignment for each read. RPKM values were computed for each ENSEMBL gene using reads mapping to exons and junctions of its longest transcript.

Processed Illumina gene expression data were downloaded from GEO and summarized to individual Ensembl IDs by taking the mean value of all probes mapped to that gene [85]. Reported probe detection values were used to remove genes from the analysis for which all probes had a value >0.05 in all samples. Affymetrix expression data were summarized using the RMA algorithm from the Bioconducter *affy *package and an updated annotation [78]. Detection calls were also generated using the MAS5 algorithm in the Bioconducter *affy *package.

To define CGI genes that were differentially expressed between normal colon and normal liver, we assayed for differential expression in processed expression microarray data using t-tests and Benjamini-Hochberg correction for multiple testing (assuming unequal variance and with a false discovery rate (FDR) of 5%). We then refined these lists to only those that had CGI promoters and had probes on the Infinium array within 200bp of their TSS that were unmethylated in normal colon (1,456 colon active and 356 colon repressed genes) before comparing methylation levels in colorectal tumors between these groups.

### Analysis of GO-terms

To analyze functional terms, Ensembl Biomart was used to map gene identifiers to GO biological process terms (Ensembl 54). Enrichment of specific terms in each gene list was then assessed using Fisher's exact test as compared to all genes present on the Infinium array. Terms that were associated with less than 10 genes on the Infinium arrays were excluded from the analysis. Data were presented as change in the percent of genes in each set as compared to the control.

### Definition of tissue specificity of gene expression

The specificity of a gene's expression pattern in normal tissues was measured using a method based on information theory [33]. A low score indicates that a gene is uniformly expressed and a high score indicates that it is expressed specifically in one tissue. For plotting, we calculated the specificity for all genes and then split them into five equally sized groups of increasing specificity. We first removed any genes from the analysis that were potentially unexpressed in all assayed samples. For microarray expression data this was done using Affymetrix MAS5 detection calls by defining genes that were 'absent' in all samples as being unexpressed in all tissues. For RNA-seq, genes unexpressed in all tissues were defined as those that had RPKM values of 0 in all tissues. The specificity of individual gene sets was examined by plotting their distribution across the five specificity groups or by plotting the scores themselves. To test significance, the specificity scores of gene sets were compared using Wilcoxon rank sum tests.

### Definition of genes mutated in breast cancer

Genes reported as mutated in breast cancer were defined using the Catalogue of Somatic Mutations in Cancer (COSMIC, [86]) database [87]. Reports of genes mutated or not mutated in breast cancer samples were downloaded from COSMIC biomart (version 52B) and used to generate two lists of genes: those mutated in at least two samples and those not mutated in any. Lists were further limited to only those genes with CGI promoters (341 mutated and 10,117 non-mutated genes). The control set for this analysis was all CGI promoter genes reported as analyzed in breast cancer by COSMIC (11,022 genes).

### Analysis of repetitive elements at promoters

To define the density of repetitive elements around TSSs, repetitive element positions were downloaded from the Repeat Masker track of the University of California, Santa Cruz (UCSC) genome browser (hg18) [88]. Custom R scripts were then used to determine whether a repeat of a given class was present in a particular genomic interval. Repeats were defined as being present if they overlapped this interval. Genes were analyzed with respect to their TSS in non-overlapping 1 kb windows upstream and downstream of the TSS (with respect to the direction of transcription). To compare gene sets we plotted the frequency of repeats found at each window within that set. Differences between sets were tested using Fisher's exact tests based upon the density of repeats within a window ± 2 kb from TSSs.

### Analysis of evolutionary conservation at promoters

We defined the level of conservation around gene promoters using two different measurements. The first was based upon the measurement of base substitutions between 17 vertebrate species [46]. To define this score, the 'aggregate' tool from the Galaxy suite of bioinformatic tools was used to generate mean Phastcons (conservation) scores in 500bp windows surrounding each TSS (using the Phastcons 17-vertebrate alignments from genome build hg18). The second score was defined using data on sequences that showed a significant depletion of short insertions and deletions in comparisons of multiple species [47]. We downloaded the locations of these sequences from the UCSC browser (hg18) [88] and then used the *coverageBed *tool from the BEDtools suite of bioinformatics tools [80] to calculate the percent of a given genomic interval occupied by these insertion and deletion purified sequences. Genes were analyzed with respect to their TSS in non-overlapping 500bp windows upstream and downstream of the TSS (with respect to the direction of transcription) by deriving mean scores for genes in the set. The significance of differences between gene sets was tested using the scores calculated for the window -2 to +2 kb from the TSS and Wilcoxon rank sum tests.

### Analysis of genes neighboring lincRNAs

We used a recent survey of lincRNAs in the human genome to define genes that had a neighboring lincRNA [49]. The nearest genic neighbor of each lincRNA was defined from that study's supplementary data and the given Refseq IDs mapped to Ensembl gene IDs. Gene sets were compared by examining the proportion of genes that had a lincRNA as their closest neighbor and significance was tested using Fisher's exact tests.

## Abbreviations

AML: acute myeloid leukemia; bp: base pair; CGI: CpG island; CM: consistently methylated; COSMIC: Catalogue of Somatic Mutations in Cancer; DNMT: DNA methyltransferases; ES cell: embryonic stem cell; GEO: Gene Expression Omnibus; GO: gene ontology; hmC: hydroxymethylated cytosine; lincRNA: long intergenic non-coding RNA; LINE: long interspersed element; LTR: long terminal repeat; MDB: methyl-binding domain; mC: methylated cytosine; PCA: principal component analysis; RPKM: reads per kb per million mapped reads; seq: high-throughput sequencing; SINE: short interspersed element; SRA: sequence read archive; TCGA: The Cancer Genome Atlas; TSS: transcription start site; VM: variably methylated.

## Competing interests

The authors declare that they have no competing interests.

## Authors' contributions

DS and RRK performed the research and analyzed data. DS, CEN, JMD, AHS, DJH, BHR and RRM helped design the research and review the manuscript. DS and RRM wrote the paper. All authors read and approved the final version of the manuscript for publication.

## Supplementary Material

Additional file 1 **1,009 hypermethylation prone genes**. Excel file containing details of the 1,009 hypermethylation prone genes in the 7 cancer types. Click here for file

Additional file 2 **Supplementary data**. PDF file containing four supplementary figures, one table and their legends. Click here for file

Additional file 3 **446 variably methylated genes**. Excel file containing details of the 446 variably hypermethylated genes along with their susceptibility in the different cancer types. Click here for file

Additional file 4 **220 consistently methylated genes**. Excel file containing details of the 220 consistently hypermethylated genes in the 7 different types of cancer. Click here for file
