# Supplementary material for: Tissue of origin determines cancer-associated CpG island promoter hypermethylation patterns
Source: Genome Biol. 2012 Oct 3;13(10):R84. doi: 10.1186/gb-2012-13-10-r84 (PMC3491412; doi:10.1186/gb-2012-13-10-r84)
Supplement: Additional file 2 — Supplementary data. PDF file containing four supplementary figures, one table and their legends. [file gb-2012-13-10-r84-S2.pdf]

Additional File 2

Supplementary Tables and Figures + Legends

List of contents:

Table S1: Promoter hypermethylation frequencies at known tumour suppressor genes

Figure S1: Methylation levels at hypermethylation prone genes vary between cancer types

Figure S2: Genes frequently hypermethylated in multiple cancer types have regulated expression patterns in normal tissues

Figure S3: Repeat densities and evolutionary conservation do not determine hypermethylation susceptibility in cancer

Figure S4: Expression patterns in normal tissues explain differential susceptibility to hypermethylation in cancer

Table S1

			Methylation Frequency in Cancer						
**Name**	**ENSEMBL gene ID**	**CpG ID (Illumina)**	**Br**	**Col**	**Pro**	**Glio**	**Lu**	**AML**	**Ovr**
CDH1	ENSG00000039068	cg11255163;cg23989635	0%	0%	0%	7%	0%	15%	0%
MLH1	ENSG00000076242	cg00893636	1%	10%	0%	0%	0%	0%	0%
BRCA1	ENSG00000012048	cg04658354;cg08993267	2%	0%	0%	0%	0%	0%	12%
RB1	ENSG00000139687	cg10552385;cg24937706	0%	0%	0%	0%	0%	0%	0%
APC	ENSG00000134982	cg16970232;cg20311501;cg21634602	28%	33%	51%	0%	44%	1%	9%

Table S1

Promoter hypermethylation frequencies at known tumour suppressor genes

We created a list of known tumour suppressor genes which when mutated in the germline are associated with pre-disposition to cancer. This was then limited to genes which had probes within CGIs and within 200bp of TSSs. Reported are these probe IDs and the % of tumours in each tissue which were methylated (mean beta > 0.3). Br=Breast, Col=Colorectal, Pro=Prostate, Glio=Glioblastoma, Lung=Lung, Ovr=Ovarian.

Figure S1

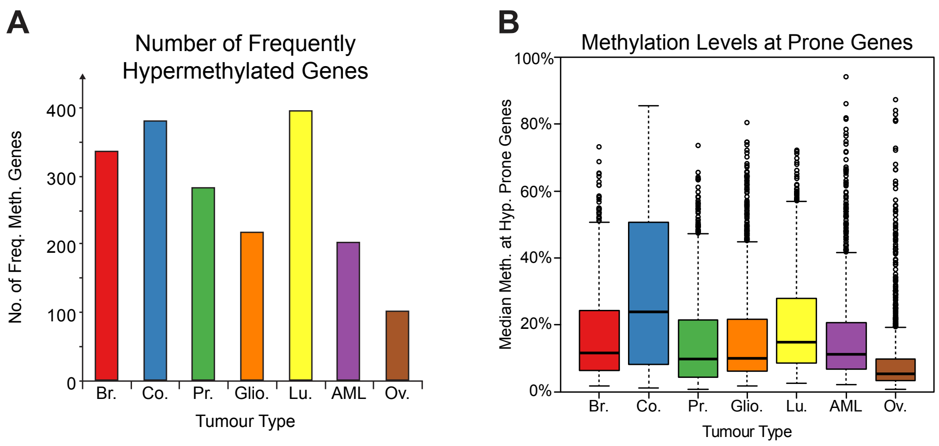
A
Number of Frequently
Hypermethylated Genes
No. of Freq. Meth. Genes
400
300
200
100
0
Br.
Co.
Pr.
Glio.
Lu.
AML
Ov.
Tumour Type
B
Methylation Levels at Prone Genes
Median Meth. at Hyp. Prone Genes
100%
80%
60%
40%
20%
0%
Br.
Co.
Pr.
Glio.
Lu.
AML
Ov.
Tumour Type

Figure S1

Methylation levels at hypermethylation prone genes vary between cancer types

A. Numbers of frequently hypermethylated genes vary between tumour types. Shown is a bargraph of the number of frequently hypermethylated genes found in each of the 7 tumour types analysed.

B. Methylation levels at hypermethylation-prone genes vary between tumour types. Shown is a boxplot of the median methylation levels found at the 1009 hypermethylation prone genes in the 7 tumour types analysed.

Figure S2

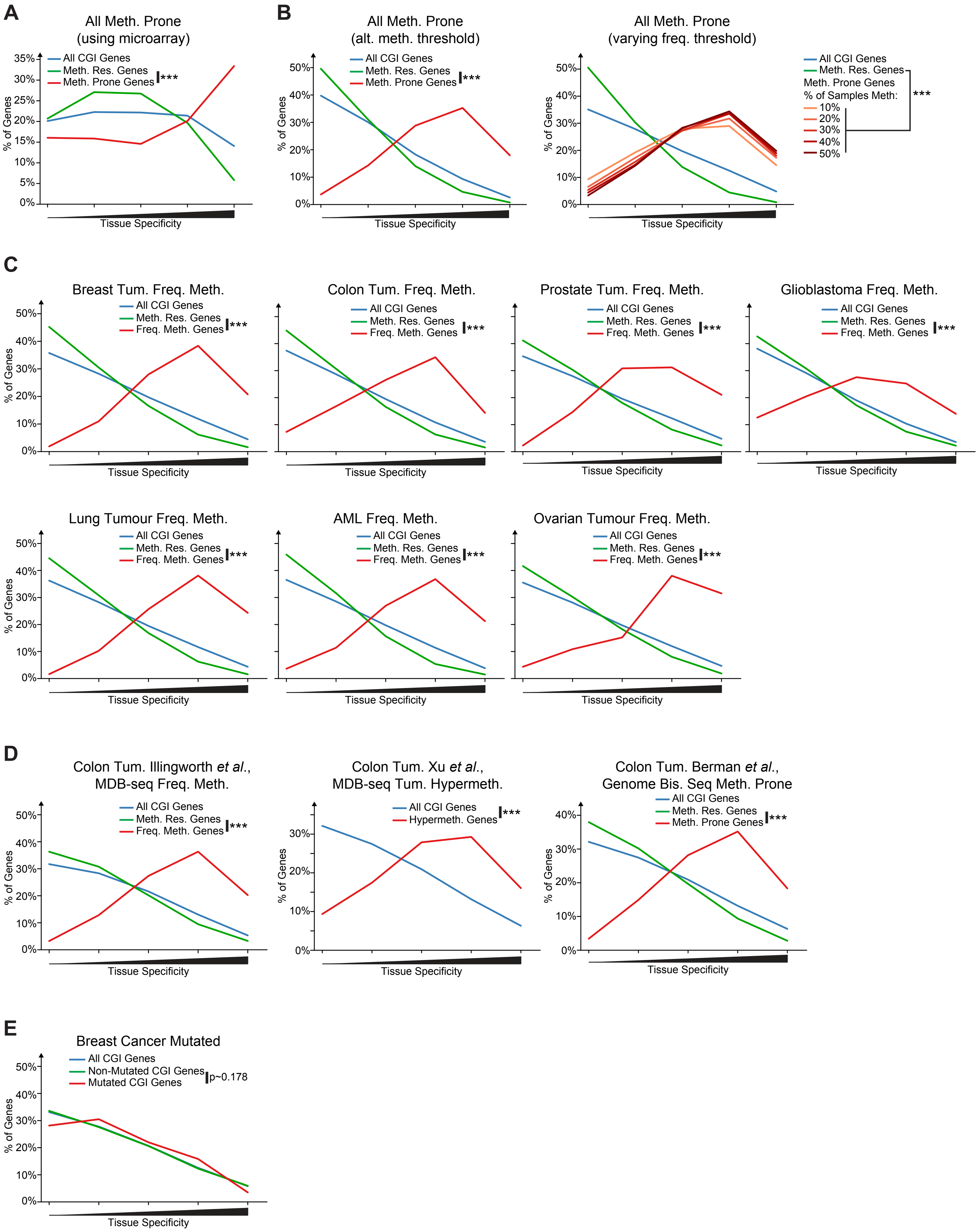

A
All Meth. Prone
(using microarray)
All CGI Genes
Meth. Res. Genes
Meth. Prone Genes
***
% of Genes
35%
30%
25%
20%
15%
10%
5%
0%
Tissue Specificity
B
All Meth. Prone
(alt. meth. threshold)
All CGI Genes
Meth. Res. Genes
Meth. Prone Genes
***
% of Genes
50%
40%
30%
20%
10%
0%
Tissue Specificity
All Meth. Prone
(varying freq. threshold)
All CGI Genes
Meth. Res. Genes
Meth. Prone Genes
% of Samples Meth:
10%
20%
30%
40%
50%
***
% of Genes
Tissue Specificity
C
Breast Tum. Freq. Meth.
Colon Tum. Freq. Meth.
Prostate Tum. Freq. Meth.
Glioblastoma Freq. Meth.
Lung Tumour Freq. Meth.
AML Freq. Meth.
Ovarian Tumour Freq. Meth.
All CGI Genes
Meth. Res. Genes
Freq. Meth. Genes
***
% of Genes
Tissue Specificity
D
Colon Tum. Illingworth et al.,
MDB-seq Freq. Meth.
All CGI Genes
Meth. Res. Genes
Freq. Meth. Genes
***
Colon Tum. Xu et al.,
MDB-seq Tum. Hypermeth.
All CGI Genes
Hypermeth. Genes
***
Colon Tum. Berman et al.,
Genome Bis. Seq Meth. Prone
All CGI Genes
Meth. Res. Genes
Meth. Prone Genes
***
% of Genes
Tissue Specificity
E
Breast Cancer Mutated
All CGI Genes
Non-Mutated CGI Genes
Mutated CGI Genes
p~0.178
% of Genes
Tissue Specificity

Figure S2

Genes frequently hypermethylated in multiple cancer types have regulated expression patterns in normal tissues

Histograms showing the distribution of tissue-specificity scores observed for different gene sets. Specificity scores for different gene sets were compared using a Wilcoxon rank sum test as indicated. (*** < 0.001). All as Figure 2A but using alternative gene sets.

A. Genes prone to hypermethylation in multiple cancer types. Gene sets are as Figure 2A but specificity scores were calculated from microarray expression data rather than RNA-seq.

B. Genes prone to hypermethylation as defined using alternative parameters. On the left the threshold used to define genes as hypermethylated was varied (see methods for details). On the right the frequency of hypermethylation required to be defined as frequently hypermethylated in a given cancer was varied.

C. Genes frequently hypermethylated in each of the individual cancer types examined in this study. Methylation resistant genes were defined as genes never methylated in that cancer type.

D. Genes aberrantly hypermethylated in colorectal cancer defined from datasets using alternative profiling methods [34-36]. Genes aberrantly hypermethylated in colorectal tumours were defined from either MBD-seq data (Illingworth *et al* and Xu *et al*) or whole-genome bisulfite sequencing (Berman *et al*). The Illingworth *et al* graph shows genes frequently hypermethylated in colon cancer, the Xu *et al* graph shows genes that are significantly hypermethylated in colon cancer and the Berman *et al* graph shows genes that are prone to hypermethylation in colon cancer (see methods for details).

E. Genes mutated or not mutated in breast cancer. Mutated genes were defined from the COSMIC database [69].

Figure S3

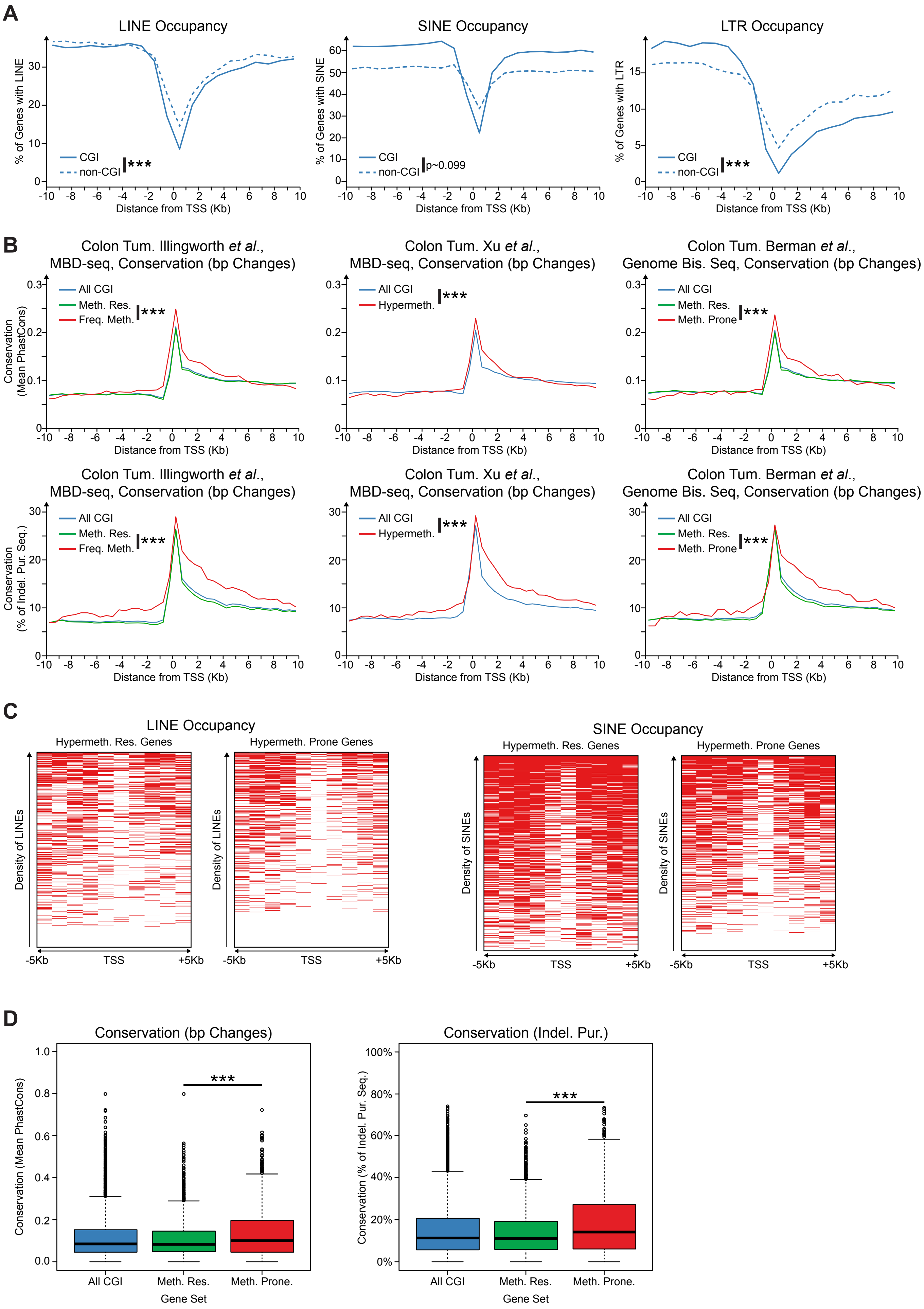
A
LINE Occupancy
% of Genes with LINE
CGI
non-CGI
***
Distance from TSS (Kb)
SINE Occupancy
% of Genes with SINE
p~0.099
LTR Occupancy
% of Genes with LTR
B
Colon Tum. Illingworth et al., MBD-seq, Conservation (bp Changes)
Conservation (Mean PhastCons)
All CGI
Meth. Res.
Freq. Meth.
Colon Tum. Xu et al., MBD-seq, Conservation (bp Changes)
Hypermeth.
Colon Tum. Berman et al., Genome Bis. Seq, Conservation (bp Changes)
Meth. Prone
Conservation (% of Indel. Pur. Seq.)
C
LINE Occupancy
Hypermeth. Res. Genes
Hypermeth. Prone Genes
Density of LINEs
-5Kb
TSS
+5Kb
SINE Occupancy
Density of SINEs
D
Conservation (bp Changes)
Conservation (Mean PhastCons)
All CGI
Meth. Res.
Meth. Prone.
Gene Set
Conservation (Indel. Pur.)
Conservation (% of Indel. Pur. Seq.)

Figure S3

Repeat densities and evolutionary conservation do not determine hypermethylation susceptibility in cancer

A. Transcriptional start sites are depleted of repetitive elements. Shown are graphs of the frequency of LINEs, SINEs and LTRs at 1Kb intervals around CGI or non-CGI TSSs (as Figure 3A).

B. Hypermethylation prone promoter regions are evolutionarily conserved. Shown are graphs of the level of conservation found in 500bp intervals around genes that are hypermethylated in colorectal tumours as defined by alternative profiling methods (as Figure 3B) [34-36]. The significance of observed differences between hypermethylated and non-hypermethylated genes was assessed using a Wilcoxon rank sum test for the scores -/+ 2Kb from the TSSs (*** $p < 0.001$).

C. Repeat densities do not determine hypermethylation susceptibility. Shown are heatmaps indicating the presence (red) or absence (white) of repeats in the region around hypermethylation prone and resistant TSSs. Repeat presence was assessed in 1Kb intervals -/+5Kb from each TSS and TSSs are ordered by their repeat density in this region.

D. Evolutionary conservation does not determine hypermethylation susceptibility. Shown are boxplots of the degree of evolutionary conservation found -/+2Kb from hypermethylation prone and resistant TSSs (measured as Figure 3B). The significance of differences between the distributions was assessed using Wilcoxon rank sum tests.

Figure S4

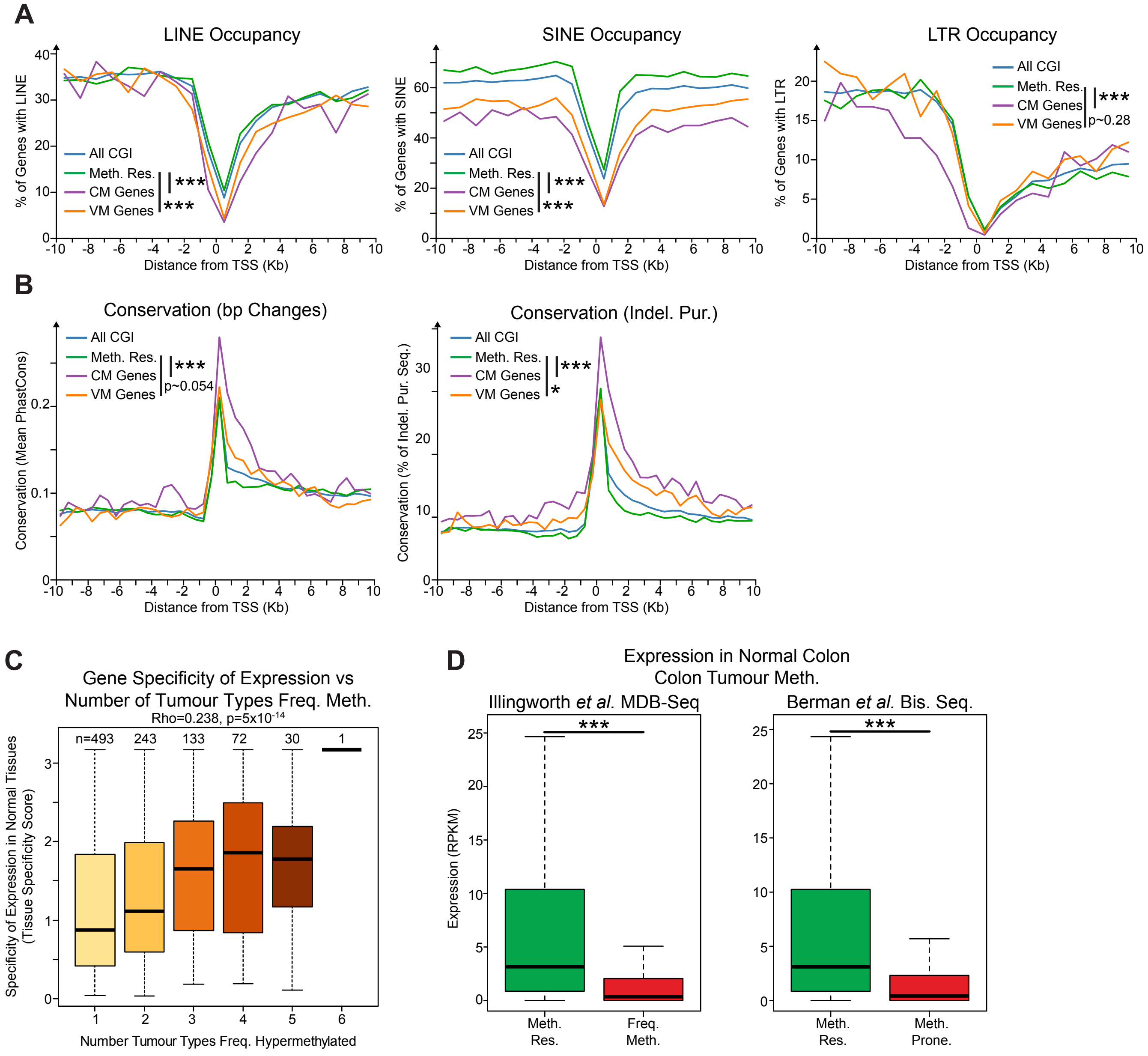
A
LINE Occupancy
% of Genes with LINE
All CGI
Meth. Res.
CM Genes
VM Genes
***
***
Distance from TSS (Kb)
SINE Occupancy
% of Genes with SINE
All CGI
Meth. Res.
CM Genes
VM Genes
***
***
Distance from TSS (Kb)
LTR Occupancy
% of Genes with LTR
All CGI
Meth. Res.
CM Genes
VM Genes
***
p~0.28
Distance from TSS (Kb)
B
Conservation (bp Changes)
Conservation (Mean PhastCons)
All CGI
Meth. Res.
CM Genes
VM Genes
***
p~0.054
Distance from TSS (Kb)
Conservation (Indel. Pur.)
Conservation (% of Indel. Pur. Seq.)
All CGI
Meth. Res.
CM Genes
VM Genes
***
*
Distance from TSS (Kb)
C
Gene Specificity of Expression vs
Number of Tumour Types Freq. Meth.
Rho=0.238, p=5x10-14
n=493
243
133
72
30
1
Specificity of Expression in Normal Tissues
(Tissue Specificity Score)
Number Tumour Types Freq. Hypermethylated
D
Expression in Normal Colon
Colon Tumour Meth.
Illingworth et al. MDB-Seq
Berman et al. Bis. Seq.
Expression (RPKM)
***
***
Meth.
Res.
Freq.
Meth.
Meth.
Res.
Meth.
Prone.

Figure S4

Expression patterns in normal tissues explain differential susceptibility to hypermethylation in cancer

A. CM and VM gene promoters are both depleted in repetitive elements. Shown are graphs of the frequency of LINEs, SINEs and LTRs at 1Kb intervals around different sets of TSSs (as Figure 3A). The significance of the differences in densities were determined using Fisher's exact tests for the repeat counts in regions -/+ 2Kb from the TSSs (*** $p < 0.001$, ** $p < 0.01$ and * $p < 0.05$).

B. CM and VM gene promoters are evolutionarily conserved. Shown are graphs of the level of conservation found in 500bp intervals around different sets of TSSs (as Figure 3B). The significance of observed differences were assessed using a Wilcoxon rank sum test based on the scores for the region -/+ 2Kb from the TSSs.

C. Specificity of expression in normal tissues is significantly correlated to the frequency of aberrant hypermethylation in cancers arising in different tissues. Shown is a boxplot of tissue-specificity scores for genes which are frequently aberrantly hypermethylated in different numbers of cancer types. A significant correlation exists between the two (Spearman's correlation, Rho=0.238, $p=5x10^{-14}$).

D. Genes hypermethylated in colon cancer are repressed in normal colon tissue. Shown are boxplots of the expression levels in normal colon of genes which are found to be aberrantly methylated or not in colorectal tumour by either MDB-seq or whole-genome bisulfite sequencing. Differences between gene groups were tested using Wilcoxon rank sum tests.
